# Fogs and Their Effects

**Published:** 1888-03

**Authors:** 


### FOGS AND THEIR EFFECTS.

It is but a small consolation to the millions of people whose respiratory function is distressed, and whose general health and comfort are interfered with by the fogs which have lately prevailed, to know that they are attributable to this or that meteorological condition of things. A marked difference is found between fogs over the sea or country and those observed in large towns, the former being, as a rule, whiter, more moist, and distinctly less irritating to the mucous membranes than the latter. This is doubtless to be explained by the fact that in towns the particles of water of which the fog is composed take up the sulphurous acid, ammoniacal vapors and other atmospheric impurities poured out by the countless chimneys of the metropolis until they give rise to almost unbearable irritation when brought into contact with the mucous membranes of the eyes, nose, and lungs. The "drier" the fog the more marked is this property, and the effects on the mortality tables is correspondingly more pronounced. The fog which enveloped London all last week in its sable mantle was remarkably moist, and therefore less irritating. When of some duration, the evil effects of the peculiar power possessed by masses of moisture to check the dissemination of vapors become very evident. The denizens of cities experience, in a more or less marked manner, all the symptoms of carbonic acid poisoning superadded to those due to a diminution in the supply of oxygen. In London, no sooner does the fog swoop down upon us than we begin to choke and experience the various unpleasantnesses incidental to partial asphyxia. The country fog is positively refreshing after a few hours in town, both morally and physically. Unfortunately, in addition to the swollen death-rates from disease we have to lament an appalling number of fatalities due directly to the interference of fog with vision and sound.—*Medical Press and Circular.*

